# Personalized dosing of nicotine replacement therapy versus standard dosing for the treatment of individuals with tobacco dependence: study protocol for a randomized placebo-controlled trial

**DOI:** 10.1186/s13063-020-04532-7

**Published:** 2020-06-29

**Authors:** Laurie Zawertailo, Christian S. Hendershot, Rachel F. Tyndale, Bernard Le Foll, Andriy V. Samokhvalov, Kevin E. Thorpe, Andrew Pipe, Robert D. Reid, Peter Selby

**Affiliations:** 1 Nicotine Dependence Services, Centre for Addiction and Mental Health, 175 College St, Toronto, Ontario M5T 1P7 Canada; 2 Department of Pharmacology and Toxicology, University of Toronto, 1 King’s College Circle, Toronto, M5S 1A8 Canada; 3 Campbell Family Mental Health Research Institute, Centre for Addiction and Mental Health, 100 Stokes St., Toronto, Ontario M6J 1H4 Canada; 4 Department of Psychiatry, University of Toronto, 250 College Street, Toronto, Ontario M5T 1R8 Canada; 5 Department of Psychology, University of Toronto, 100 St. George St., Toronto, Ontario M5S 3G3 Canada; 6 Department of Family and Community Medicine, University of Toronto, 500 University Ave, Toronto, Ontario M5G 1V7 Canada; 7 Addictions Division, Centre for Addiction and Mental Health, Toronto, Ontario, 100 Stokes St., Toronto, Ontario M6J 1H4 Canada; 8 Institute for Mental Health Policy Research, Centre for Addiction and Mental Health, 33 Russell St, Toronto, Ontario M5S 2S1 Canada; 9 Department of Psychiatry, McMaster University, 100 West 5th, Hamilton, Ontario L8N 3K7 Canada; 10 Dalla Lana School of Public Health, 155 College St., Toronto, Ontario M5T 3M7 Canada; 11 The Applied Health Research Centre, Li Ka Shing Knowledge Institute, St. Michael’s Hospital, 250 Yonge St., Toronto, Ontario M5G 1B1 Canada; 12 University of Ottawa Heart Institute, 40 Ruskin St., Ottawa, Ontario K1Y 4W7 Canada

**Keywords:** Nicotine replacement therapy, Smoking cessation, Dose titration, Nicotine patches, Placebo patches

## Abstract

**Background:**

Medications for smoking cessation are currently only effective in helping a minority of smokers quit. Drug development is slow and expensive; as such, there is much interest in optimizing the effectiveness of existing treatments and medications. Current standard doses of nicotine replacement therapy are not effective for many smokers, and in many cases, the amount of nicotine provided is much less than when a smoker is smoking their usual number of cigarettes. The proposed study will test if titrating the dose of the nicotine patch (up to 84 mg) will improve quitting success compared to those receiving a 21-mg nicotine patch with increasing doses of placebo patch.

**Methods:**

This is a multicenter, pragmatic, two-arm, placebo-controlled, block randomized controlled trial. We will recruit participants who smoke at least 10 cigarettes daily and are interested in making a quit attempt. After 2 weeks of usual treatment with a 21-mg patch, participants who fail to quit smoking (target *n* = 400) will be randomized to receive escalating doses of a nicotine patch vs matching placebo patches for an additional 10 weeks or up to a maximum dose of 84 mg per day. Those who stop smoking during the first 2 weeks of usual treatment will continue with 21 mg patch treatment for 10 weeks and will form an additional comparison arm. In addition to the medication, participants will receive brief behavioral counseling at each study visit. The primary outcome will be biochemically confirmed continuous abstinence from smoking during the last 4 weeks of treatment (weeks 9 to 12).

**Discussion:**

Research evidence supporting the effectiveness of personalized doses of nicotine replacement therapy could change current practice in a variety of healthcare settings. Given the evidence that quitting smoking at any age diminishes the risk of tobacco-related morbidity and mortality, even small increases in absolute quit rates can have a substantial population-level impact on reducing smoking-related disease, mortality rates, and associated healthcare costs.

**Trial registration:**

ClinicalTrials.gov, NCT03000387. Registered on 22 December 2016.

## Background

The large decline in smoking prevalence over the last 50 years has slowed considerably such that 1 in 6 (4.6 million) Canadians still smoke tobacco [1]. However, cigarette smoking remains a leading cause of preventable death in Canada [2] and around the world accounting for more than 4 million deaths each year worldwide [3]. It should be noted that the harm from smoking is attributed to the thousands of chemicals and 60 known carcinogens in combusted tobacco and not nicotine per se [4]. Therefore, in Canada, all formulations and strengths of nicotine replacement therapy (NRT; a 24-h sustained-release transdermal patch, and immediate-release gum, lozenge, inhaler, and oral spray) are available without a prescription because they pose a minimal risk even with long-term use [5]. NRT is the most commonly used medication to help quit smoking [6] and is safe, even in smokers with cardiovascular disease [7, 8]. In light of its superior safety profile, Health Canada mandated a label change in 2013 advising “thorough consideration” of NRT before prescribing either varenicline or bupropion to any smoker, further stating that “in many cases, nicotine replacement therapy should be tried before prescribing varenicline or bupropion” [9], emphasizing the need to optimize the effectiveness of this first-line medication. Given the clinical significance of even small effect sizes associated with quitting [10], optimizing the use and efficacy of the nicotine patch is a pragmatic and feasible approach to the continuing problem of tobacco use disorder in the current, hard-to-treat population of smokers.

In controlled clinical trials, NRT increases smoking cessation rates at 1-year follow-up by approximately 50–60% over placebo [11], but its real-world effectiveness is lower [12, 13]. The nicotine patch is the most commonly used [6] form of NRT but is less effective in heavy smokers (≥ 20 cigarettes/day) presumably due to inadequate replacement of nicotine to treat withdrawal, suggesting that higher doses may be more effective [14, 15]. However, studies have not found an advantage for a fixed high-dose nicotine patch over standard doses (42/44 mg vs. 21/22 mg) [16]. However, fixed doses can be ineffective because in some cases the dose will exceed an individual’s level of tolerance to nicotine, causing symptoms of toxicity, while for others it will be inadequate to eliminate withdrawal and cravings [17]. These individual variations in nicotine tolerance are at least in part due to the pharmacogenetics of Cytochrome P450 2A6 (CYP2A6), the hepatic enzyme primarily responsible for nicotine metabolism [18]. Personalizing the dose by adding patches as tolerated until cessation is achieved can address both of these shortcomings.

Furthermore, human experimental studies demonstrate that smoking behavior can be extinguished with adequate doses of intravenous nicotine [18] or a patch [17]. The aim of this study is to evaluate the effectiveness of personalizing transdermal nicotine patch dose using a titration protocol designed to fully replace nicotine obtained from an individual’s smoking and compare this to standard treatment (21 mg patch) plus titration with placebo patches.

### Study objectives and hypothesis

Our goal is to develop empirically validated, safe and effective, personalized treatment for smokers trying to quit who do not respond to standard treatment. The main objective of this clinical trial is to determine the efficacy of 10 weeks personalized dosing of the nicotine patch in motivated smokers unable to quit during the first 2 weeks of standard-dose patch use (21 mg). Therefore, we designed a multicenter, 12-week, randomized, placebo-controlled, double-blind study to compare the effectiveness of personalized doses of the nicotine patch (maximum of 84 mg/day) to standard 21 mg patch treatment in treatment-seeking adult smokers.

We hypothesize that a personalized dose of a nicotine patch will increase cessation rates relative to standard dosing based on the following empirical evidence. First, standard doses of a nicotine patch have low absolute efficacy due in part to inadequate replacement [11, 19]. Second, a fixed high-dose patch (44 mg) is no better than standard therapy in rapid metabolizers of nicotine unless there is a higher percent replacement of baseline nicotine levels [20, 21]. Third, using a nicotine patch prior to one’s target quit date (preloading), which allows for smoking concurrently with NRT, is safe and is associated with higher quit rates [22]. Fourth, our exploratory studies to date and clinical practice have indicated that titration with a nicotine patch up to 84 mg is associated with quitting completely or achieving a > 50% reduction confirmed by exhaled carbon monoxide (CO) readings, with reduced cravings and increased aversion to cigarettes [23]. Lastly, a nicotine patch has negligible abuse liability and dependence potential due to its slow onset and long duration of action [24]. Furthermore, clinical trials have shown that standard NRT doses are inadequate for rapid metabolizers of nicotine (i.e., those who inactivate nicotine to cotinine and then to 3-hydroxycotinine [3-HC] more quickly than others due to genetic variation affecting the activity of the hepatic enzyme CYP2A6) [20, 25]. A recent pilot study indicated rapid metabolizers may respond better to higher transdermal NRT doses [21]. Therefore, we aim to test a secondary hypothesis that baseline nicotine metabolite ratio (NMR) will moderate the experimental treatment effect, such that the benefit of dose titration will be greater in those participants who are rapid metabolizers of nicotine, and the final titrated dose they reach will be higher on average than the slow metabolizers. We further hypothesize that slow nicotine metabolizers will be more likely to quit on the standard 21 mg dose and stay in group C compared to rapid metabolizers. NMR will then be tested as a predictor of who will benefit most from the dose titration approach. This will be useful for optimization of treatment resources and cost effectiveness.

## Methods/design

### Study design

**Fig. 1:**
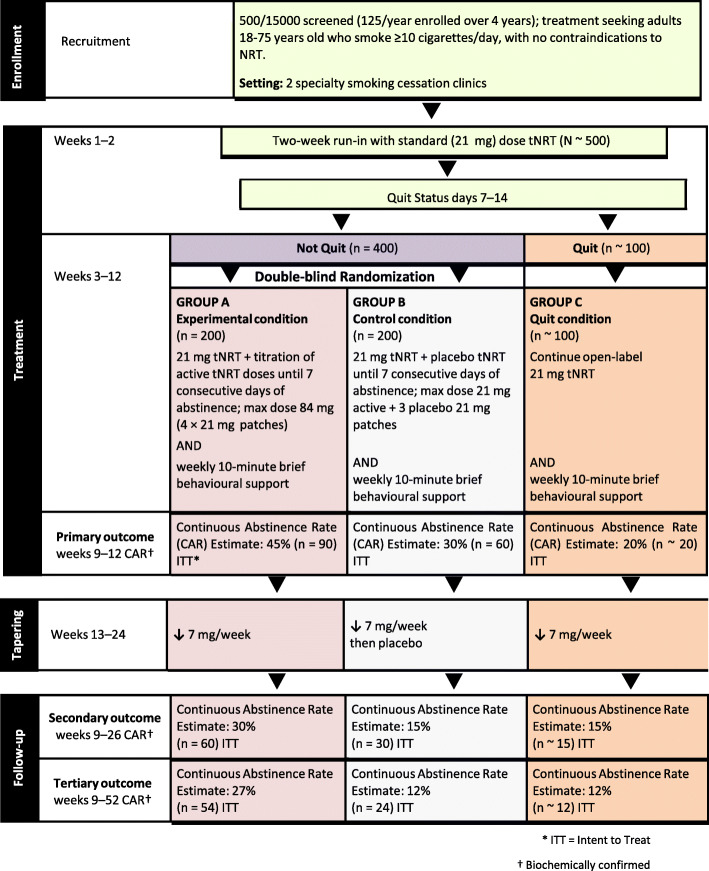
Flow diagram of proposed study

This clinical trial will use a double-blind, placebo-controlled, dose escalation design, with random assignment of participants to an experimental (group A) and a control condition (group B) for 10 weeks after failure to achieve abstinence during an open-label run-in period of approximately 2 weeks using a 21-mg nicotine patch daily. Those who stop smoking during the run-in phase will continue with open-label nicotine patch 21 mg/day (group C). See Fig. 1 for more details.

For the purpose of randomization following the 2-week run-in with the 21-mg nicotine patch, quit status will be determined by participants’ self-reported 7-day point prevalence abstinence (PPA), defined as no smoking, not even a puff, for the past 7 days. Reports of abstinence will be confirmed by exhaled CO of less than 5 ppm.

In the event of an emergency and/or a situation where the safety, care, and best interest of the participant is compromised by not knowing the treatment allocation, the QI or PI will contact the study pharmacy staff to initiate unblinding procedures. The unblinded pharmacist will retrieve, open, and communicate the information on the participant’s unblinding/randomization slip. The slips will be kept in a tamper-proof, opaque envelope in a locked cabinet on site.

### Inclusion criteria

Eligible participants will be treatment-seeking daily tobacco smokers (at least 10 cigarettes per day), aged 18 to 75 years, interested in using a nicotine patch for smoking cessation, and intending to make a quit attempt within the next 30 days.

### Exclusion criteria

Exclusion criteria include as follows: at least weekly use of tobacco products other than cigarettes (e.g., oral tobacco, e-cigarettes); breastfeeding, pregnancy or not using a reliable form of birth control; any generalized skin disorders precluding use of the patch; any known hypersensitivity or allergies to any of the components of the nicotine patch; any life-threatening arrhythmias or severe/worsening angina pectoris; myocardial infarction or cerebral vascular accident in the past 2 weeks; currently using or has used NRT or other smoking cessation pharmacotherapy within the past 2 weeks; current (in the past month) active substance dependence (excluding caffeine) or unstable psychiatric condition which would compromise study compliance; diagnosed with a terminal illness; current regular use of e-cigarettes or other vaping devices containing nicotine (and not willing to stop using these devices for the duration of the study).

### Study settings

Study settings include a smoking cessation treatment clinic at the Centre for Addiction and Mental Health (CAMH) in Toronto, Canada, and a smoking cessation treatment clinic at the University of Ottawa Heart Institute (UOHI) in Ottawa, Canada.

### Recruitment and retention

Participants will be recruited from the community and the treatment clinics at the two study sites. They will be self-identified treatment-seeking smokers willing and able to participate for the full duration of the study. The goal is to enroll a cohort most likely to represent real-world smokers who want to stop smoking using a nicotine patch and for whom it will be safe to do so. Recruiting from two sites will further improve the generalizability of our findings to the larger population of treatment-seeking smokers, while also ensuring we meet recruitment targets. We will employ internal and external recruitment strategies approved by each hospital’s research ethics board including pamphlets, posters, radio advertising, and social media posts.

In addition to the cost-free medication and behavioral counseling, participants will be compensated for their time during study participation. Specifically, compensation worth $10 (gift card or cash) plus travel reimbursement (in the form of a parking fee, bus tickets, etc.) will be provided for each in-person visit from study weeks 3 to 13. At the initial assessment visit (week 0), participants will receive travel reimbursement but no monetary compensation. Finally, participants who attend the long-term follow-up in-person visits (weeks 26 and 52) will receive compensation worth $25 (gift card or cash).

### Pre-screening

Potential participants who are either self- or practitioner-referred will participate in a pre-screening assessment conducted by research staff on the telephone or in person. Potential participants will be invited for an initial assessment (if eligible for the study) or referred to other available tobacco use disorder treatment (if not eligible for the study).

### Initial assessment

At the initial assessment clinic visit (~ 3 h), written informed consent will be obtained from potential participants by the research analyst prior to any data acquisition. They will then complete a series of self-administered forms (Drug Abuse Screening Test [DAST-10] [26], Depression, Anxiety and Stress Scale [DASS 21] [27], Fagerström Test for Nicotine Dependence [FTND] [28], Alcohol Use Disorders Identification Test [AUDIT] [29], 36-Item Short-Form Health Survey [SF-36] [30, 31], Questionnaire of Smoking Urges - Brief [QSU-brief] [32, 33], Patient Health Questionnaire [PHQ-9] [34], Minnesota Nicotine Withdrawal Scale [MNWS] [35], and modified Cigarette Evaluation Questionnaire [mCEQ] [36]) and staff-administered forms (demographic questions, history of smoking and quit attempts, social exposure to smoking, substance use, medical history and medical assessment, including vital signs, height and weight and concomitant medication record). Participants will also undergo a physical examination, urine pregnancy test (if applicable; e.g., female, pre-menopause), and exhaled CO test and will provide a venous blood sample (10 ml) to assess 3-HC/cotinine ratio (NMR). The participant will provide an additional 10 mL of blood if they consented to the genetics sub-study. Participants may elect to take part in the genetics sub-study at any point during the study. The samples will be collected, processed (plasma aliquot, whole blood transferred to polypropylene tube), and stored in a freezer (acceptable freezing temperature − 80 to −20 °C) prior to being transported to the laboratory for analysis. Please see the SPIRIT checklist (Additional file 1) for all of the assessment and visit procedures.

### Run-in phase (weeks 1 and 2)

At the end of the initial assessment, eligible participants will start their participation in the run-in phase. Participants will receive a 2-week package (14 units) of 21-mg nicotine patches, will be instructed to start using the medication on their target quit day (determined at the initial assessment), and will be asked to abstain from smoking. The participant will also receive brief behavioral support and check-in by telephone at week 2. Participants will be asked to bring all used and unused patches to their subsequent clinic visits to monitor compliance.

#### Back-up nicotine patches

All participants will be provided 3 days’ worth of standard-dose (21 mg) nicotine patches to be used in the case of an emergency preventing them from attending their scheduled appointment. The back-up nicotine patches will be labeled by pharmacy to differentiate them from the prescribed treatment patches. These patches will be replenished if the used back-up nicotine patches are returned.

### Randomization

After the 2-week run-in phase, stage 2 will be initiated, in which participants who fail to achieve 7-day abstinence (self-report of not smoking for at least the past 7 days at week 3 study visit) will be randomly assigned to the experimental (group A, escalating active patches) or a control condition (group B, escalating placebo patches) for 10 additional weeks of treatment. To maintain allocation concealment, participants will be randomized 1:1 in permuted blocks of varying sizes. The randomization schedule will be generated by the research pharmacist under guidance of the co-investigator statistician and stored securely in the pharmacy. Only the research pharmacists will be unblinded to group assignment. Participants, investigators, and research staff will be blind to group A or B assignment. Participants who stop smoking during the run-in phase will not be randomized but instead will continue with an open-label nicotine patch 21 mg/day (group C) for another 10 weeks. We estimate that 20% of participants will be able to quit smoking in the first 2 weeks to comprise group C. This group will be used to test some of the exploratory hypotheses outlined above.

### Titration phase (weeks 3–7)

Participants in groups A and B will receive an active nicotine patch (21 mg/day) for the entire titration phase in addition to escalating doses of either active patches (group A) or placebo patches (group B) per the following dose titration protocol. The nicotine patch will be dispensed weekly during this titration phase, with dose adjustments made by the study physician at each weekly study visit based on tolerability and the average number of cigarettes smoked per day (CPD) in the preceding week (see Table 1 for the dosing algorithm based on amount of continued smoking). If adverse effects are experienced at a given dose, a dose reduction back down to a previously tolerated dose will be permitted as determined by the study physician.

**Table 1:** Algorithm for weekly dose titration based on amount of smoking during the previous week

If participant reports smoking…	Add this patch to current dose
0 cigarettes	Continue with last weeks’ dose
1–5 cigarettes per day	Add 7 mg patch (or placebo) to last week’s dose
6–9 cigarettes per day	Add 14 mg patch (or placebo) to last week’s dose
10 or more cigarettes per day	Add 21 mg patch (or placebo) to last week’s dose

Group C will continue to visit the clinic on a bi-weekly basis (weeks 3, 5, and 7) and receive 21 mg/day of a nicotine patch, and telephone follow-up visits in the intervening weeks (weeks 4 and 6). No short-acting NRT will be permitted for any participant. Participants will be contacted by telephone 1 to 2 days in advance of their scheduled clinic visits during the titration phase to remind them of their upcoming appointment.

### Maintenance phase (weeks 8–12)

**Fig. 2:**
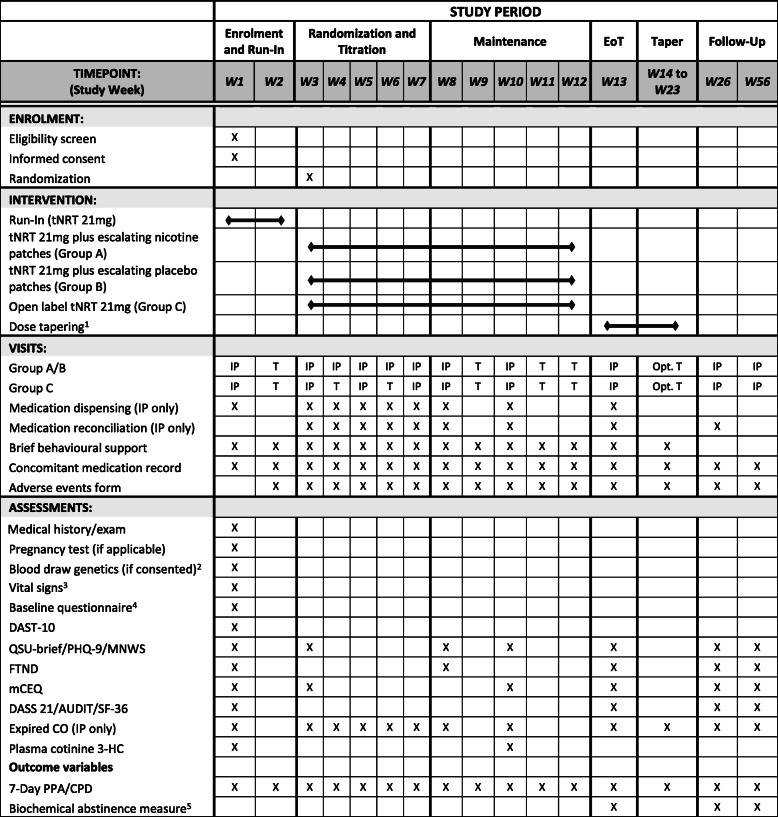
Schedule of enrolment interventions and assessments. ^1^Decrease by 7 mg/week—the duration of taper will depend on the dose at week 12; ^2^Blood for genetics sub-study may be drawn at any week; ^3^Sitting systolic/diastolic BP, pulse, respiratory rate, and body temperature were measured; ^4^Demographics, readiness, and importance to stop smoking, smoking behaviors; ^5^Urine anabasine (tobacco-specific biomarker) at week 13 (EoT) and urine cotinine at weeks 26 and 52. List of abbreviations: 7-day PPA = 7-day point prevalence abstinence; AUDIT = Alcohol Use Disorders Identification Test; CO = carbon monoxide; CPD = cigarettes per day; DASS 21 = Depression, Anxiety and Stress Scale; DAST-10 = Drug Abuse Screening Test; EoT = end of treatment visit; FTND = Fagerström Test for Nicotine Dependence; IP = in person; mCEQ = cigarette evaluation questionnaire; MNWS = Minnesota Nicotine Withdrawal Scale – validated measure of tobacco withdrawal during cessation treatment; Opt = optional; PHQ-9 = Patient Health Questionnaire—validated self-completed measure of depressive symptoms mapped on to DSM-IV criteria; QSU-brief = 10-item Questionnaire of Smoking Urges; SF-36 = 36-Item Short-Form Health Survey; T = telephone; tNRT = transdermal nicotine replacement therapy

At week 8, the dose titration phase will end and participants will be maintained on the patch dose achieved at the end of the titration phase for the remainder of the study in order to determine the primary outcome of sustained (continuous) abstinence from weeks 9 through 12 (assessed at study visits 10 through 13). All participants will attend a clinic visit at week 8 to receive 2 weeks of patches (at their current dose) and brief behavioral support, and exhaled CO will be measured to confirm self-reported smoking status. Participants will be contacted by telephone at week 9 for brief behavioral support and to complete weekly assessments (self-reported smoking status, adverse events, and concomitant medication). At the week 10 visit, all participants will return to the clinic to receive 3 weeks of patches (at their current dose) to last until the end of the treatment phase. At this visit, participants will also receive brief behavioral support, provide a venous blood sample (10 ml) to determine plasma cotinine levels, undergo an exhaled CO test, and complete several questionnaires (see Fig. 2).

### Tapering phase (weeks 13 onward)

At this end-of-treatment visit, participants will provide a urine sample to assess urinary anabasine (a marker of smoking in the presence of NRT) [37]. In addition to self-reported smoking status and CO measures taken at each visit, urinary anabasine and anatabine concentration will be used as the principle objective measure of the primary outcome (continuous abstinence from smoking during the last 4 weeks of treatment). We will use the established criteria set out by Jacob et al. where both anabasine and anatabine need to be present in concentrations > 2 ng/ml to classify a person as a tobacco user [38]. The samples will be stored in a freezer (− 80 to − 20 °C) prior to being sent to a laboratory for analysis. At this final visit, participants will receive the appropriate amount of patches (depending on their dose at the time) with clear instructions for tapering their patch dose down by 7 mg/week. In all groups, the known 21 mg NRT patch will be reduced first, followed by the study patches (may be real or placebo). Alternatively, participants who have not achieved continuous abstinence during weeks 9–12 of the study may choose to be transferred into clinical care to continue with their quit attempt. Participants may be contacted for a telephone follow-up during this phase, if requested by the participant or physician, to assess progress and smoking status.

### Weekly behavioral support and assessments (weeks 1–13)

Participants in all groups will receive brief behavioral support weekly and be asked about their smoking status, adverse events, and concomitant medication. This will occur either at clinic visits or via telephone visits.

### Post-treatment follow-up

Follow-up clinic visits will occur at weeks 26 and 52 (6 and 12 months following study enrolment) to assess sustained long-term abstinence [39]. Participants will undergo a urinary cotinine test (to detect any nicotine use), an exhaled CO test, and complete self-administered questionnaires (DASS 21, FTND, AUDIT, SF-36, QSU-brief, PHQ-9, MNWS, mCEQ, and concomitant medication record).

### Trial management

Under the supervision of the qualified investigator (QI) at each site, research staff will be responsible for day-to-day conduct of the trial. The research staff (one research analyst at each site) will handle all administrative aspects of the trial and day-to-day operations including screening, scheduling, study visits, telephone contacts, providing brief behavioral support, and handling of data. The principal QI (co-author PS) will monitor adverse events across the two study sites on a bi-weekly basis. All serious unexpected adverse drug reactions will be reported to Health Canada as per the requirements of a regulated clinical trial.

### Data management

All participant data will primarily be stored on a secure online database hosted by Research Electronic Data Capture (REDCap RCT). Each participant will also have a paper file that will be stored in a locked filing cabinet behind two locked doors. Files will be labeled with participant ID numbers and will not include the participant’s name. At a minimum, these paper files will include the following forms: original Participant Eligibility Checklist with a signed statement of eligibility by the Principal Investigator or Delegate; Medical Assessment Form; Nursing Assessment Form; baseline PHQ-9; Initial Assessment Checklist; Telephone Visit Checklist; In-Person Visit Checklist; Adverse Clinical Events Form; Concomitant Medication Record. All data captured on these paper forms will also be entered into the study’s secure online REDCap database. Additionally, in the case of technical difficulties precluding the use of the web-based database at the time of an in-person visit, necessary data will be collected on the appropriate paper forms, which will be entered into the REDCap database upon resolution of the technical difficulty. Any such paper assessment forms will be stored in the participant’s paper file as the source document. To document progress and research contact with each participant, a Research Note will be written in each participant’s electronic health record following each in-person study visit.

The original/paper signed consent form(s) will be stored in the “Consent Binder” in a locked cabinet, separately from other participant source documentation. A scanned copy of the consent form is uploaded to the participant’s electronic medical record (EMR).

### Monitoring

The study monitor will conduct monitoring visits at each site according to the following schedule: the Study Initiation Visit will occur at each site after obtaining all regulatory approvals (REB, NOL, etc.) but prior to enrolment of the first research participant; the first monitoring visit will be done after the first 5 participants have been enrolled at the site; the second monitoring visit will be done after an additional 20 participants have been enrolled at the site; additional monitoring visits will occur annually or after every 30 additional participants have been enrolled at the site, whichever comes first; the study closeout visit will be after the last participant has completed his/her involvement in the study (participant data not verified from previous visit should be completed during this visit). At each of these visits, the study monitor will review all of the source data in the CRF for 10% of the research participants. Should issues regarding data be observed during a monitoring visit, the data from all participants may be verified.

Since there are no early stopping rules or planned interim analysis and because the medication being investigated in the study is available on pharmacy shelves, a Data Monitoring Committee was deemed unnecessary.

### Outcome measures

#### Primary outcome measure

The primary outcome measure will be continuous abstinence from tobacco use during weeks 9–12 (as reported at clinic visits on weeks 10 and 13, and telephone interviews on weeks 11 and 12). Participants will be classified as abstinent if they respond “no” to both of the following questions: (i) “have you smoked any cigarettes (even a puff) in the last 7 days?” and (ii) “Have you used any tobacco product (i.e., chew, snuff, pipe, cigar, etc.) in the last 7 days?” Self-report will be biochemically confirmed with expired CO at each in-person visit and urinary anabasine at week 13.

#### Secondary outcome measures

The secondary outcome measures are self-reported continuous abstinence through week 9 to weeks 26 and 52, confirmed by urinary cotinine < 200 ng/ml (assay limit of detection) at the 26- and 52-week follow-up visits.

To test the hypothesis that rates of nicotine metabolism moderate the effect of patch titration, we will compare the quit outcomes in rapid versus slow nicotine metabolizers within and among the treatment groups.

### Statistical analysis

Descriptive statistics will be used to summarize baseline characteristics. Baseline characteristics will be examined for any statistically significant differences between randomized groups. Such variables will be used as covariates in an adjusted analysis. The primary analysis will follow the intention-to-treat principle (ITT) [40] whereby all participants that drop out of the study or are otherwise lost-to-follow-up will be classified as still smoking. The primary analysis will compare the proportions of patients in each group (group A vs group B) to achieve smoking cessation using Pearson’s chi-square test. Additional analysis will employ logistic regression to adjust for variables known to be strongly associated with smoking cessation, including but not limited to age, sex, and psychiatric comorbidities.

#### Analysis of secondary and tertiary outcomes

The proportion of patients who remain abstinent from week 9 to weeks 26 and 52 will be compared by logistic regression, weighted by the inverse probability of being included in the subset. Exploratory analysis of the role of nicotine metabolism on quit outcome will be performed: (1) initial analysis will compare the proportions of patients with rapid or slow NMR who achieve the primary outcome within each treatment group using a chi-square; (2) we will then conduct a post hoc analysis of NMR as a predictor or moderator of treatment outcome in the two treatment groups; and (3) an exploratory analysis of the correlation between final NRT dose given and NMR.

#### Sample size calculations

The anticipated absolute continuous abstinence rate for group A (weeks 9 to 12) for the primary outcome measure is set at 45%. This is based on the quit rates reported for combination NRT (nicotine patch and short-acting NRT) [16]. Trials of combination NRT were used to inform this estimate because: (1) randomized trials of a high-dose nicotine patch are not available to inform the projected treatment size, and (2) similar to nicotine patch, combination NRT also allows upward titration to reach satiety. It is anticipated that the abstinence rates for an active titrated nicotine patch will at least meet, if not exceed those of combination NRT. The estimated continuous abstinence rates during weeks 9 to 12 for group B are 30% based on published literature of the effects of a standard nicotine patch in clinical trials [41]. Assuming *p* = .05, a sample of 163 per group provides 80% power to detect this difference at weeks 9 through 12. With an estimated 20% quit rate during the 2-week run-in period, we therefore plan to recruit approximately 500 participants into the trial until we have 400 eligible for randomization. Randomized groups of 200 participants per group will ensure that the group is adequately powered for longer-term outcomes as well. Since we are using ITT, all participants randomized to a treatment arm will be included in the analysis so our *n* will remain constant and our power to detect a difference between groups should not be significantly affected by loss to follow-up as long as it is more or less equal in the two arms. Our overall proportion of participants defined as quit will decrease but the difference between the two groups should remain the same.

### Ethics and dissemination

The study has been approved by the hospital Research Ethics Boards (REB) at both study sites. Protocol amendments are submitted to each hospital REB for approval prior to implementation. Once approved, the amendment is submitted to Health Canada in compliance with procedures for regulated trials. If the protocol change necessitates revisions to the consent form, all participants currently enrolled in the study will be told of the changes and asked to sign the new version of the consent form. All REB-approved documents are version controlled and stored in the investigator study binder (ISB) with a corresponding log of amendments.

Trained study staff (research analyst) will conduct the informed consent procedure and obtain written informed consent as per Good Clinical Practice guidelines prior to initiating any other study-related activities. The research analyst will provide the participant with the study consent form (Additional file 2) to read carefully, or have it read to them if applicable. Once the participant has read through the consent form, the study staff will prompt the participant for any questions, concerns, or comments. Participants that consent to the main study will be given the opportunity to participate in the Genetics sub-study (separate consent form; Additional file 3). Participants may elect to take part in the Genetics sub-study at any point during their participation in the main study. Participation in the sub-study includes collecting one venous blood sample (10 ml). The blood sample will be labeled with a UPC code and no other identifying information stored in a locked freezer. Genotype analyses of nicotine metabolizing enzymes and other genes related to smoking cessation treatment outcomes will be conducted.

All personal health information collected for study purposes will be stored securely as per Good Clinical Practice guidelines. Original copies of the Informed Consent documentation will be stored in a locked filing cabinet behind two locked doors. This documentation will be kept separate from all other study-related documents. Only the sponsor (CAMH) will have full access to the final trial dataset. All data and results from the subsite (UOHI), excluding PHI, are owned by CAMH.

#### Dissemination

At the completion of the study, the PI will share the results through academic presentations and publication in peer-reviewed journals. Findings will be incorporated into Canadian clinical smoking cessation practice guidelines. In addition, results will be available to view on clinicaltrials.gov.

## Discussion

Fixed doses of NRT can be ineffective for many patients as the given dose can exceed some individuals’ level of tolerance to nicotine while for others it may be an inadequate dose that fails to sufficiently quell withdrawal and cravings [23]. By personalizing doses of NRT, clinicians may be able to assist individuals on either end of the dosing spectrum. The aim of this study is to understand the efficacy of 10 weeks of personalized dosing of a nicotine patch in motivated smokers unable to quit after 2 weeks of a standard nicotine patch (21 mg). The study optimizes the current evidence-based practice for smoking cessation treatment (NRT with brief behavioral counseling) using a personalized dosing approach. Given the pragmatic nature of the study and the heterogeneity of the participants, the results from this study will be externally valid and will be applicable to a wide variety of healthcare settings.

The challenges encountered to date with the implementation of this investigator-initiated study have been primarily related to the lack of ready access to matching placebo patches that satisfy the safety and manufacturing requirements of clinical trial regulators in Canada. In addition, although the design is pragmatic to include those with psychiatric or substance use comorbidities, trial burden (weekly visits, adherence to the protocol) might make retention problematic for these participants. Concurrent cannabis smoking and alcohol consumption could affect the primary outcome and the outcome measure had to be chosen to limit false positives.

Given the strong link between smoking, cancer, and cardiovascular disease and evidence that quitting smoking at any age diminishes this risk, even small increases in absolute quit rates can have a substantial population-level impact on reducing the incidence of smoking-related disease, mortality rates, and associated healthcare costs.

## Trial status

The original trial protocol was first approved by the hospital Research Ethics Board on June 9, 2015, and by Health Canada Natural Products Directorate on December 12, 2016. The trial was registered on clinicaltrials.gov on December 22, 2016. The trial is currently ongoing as protocol version 8.0, dated October 9, 2019, approved by the hospital Research Ethics Board on October 21, 2019. Recruitment for both sites began in January 2018 and is expected to be completed by January 2022.

## Supplementary information

**Additional file 1.** SPIRIT checklist.

**Additional file 2.** Study consent form.

**Additional file 3.** Genetics sub-study consent form.

## Data Availability

The datasets generated or analyzed during the current study are available from the corresponding author on reasonable request.

## Abbreviations

3-HC: 3-Hydroxycotinine
AUDIT: Alcohol Use Disorders Identification Test
CAMH: Centre for Addiction and Mental Health
CPD: Cigarettes per day
CO: Carbon monoxide
CYP2A6: Cytochrome P450 2A6
DASS 21: Depression, Anxiety and Stress Scale
DAST-10: Drug Abuse Screening Test
FTND: Fagerström Test for Nicotine Dependence
ITT: Intention-to-treat principle
mCEQ: Modified Cigarette Evaluation Questionnaire
MNWS: Minnesota Nicotine Withdrawal Scale
NMR: Nicotine metabolite ratio (3-HC/cotinine)
NRT: Nicotine replacement therapy
UOHI: University of Ottawa Heart Institute
PHQ-9: Patient Health Questionnaire
PPA: Point prevalence abstinence
QI: Qualified investigator
QSU-brief: Questionnaire of Smoking Urges - Brief
SF-36: 36-Item Short-Form Health Survey
