# Supplementary material for: Personalized dosing of nicotine replacement therapy versus standard dosing for the treatment of individuals with tobacco dependence: study protocol for a randomized placebo-controlled trial
Source: Trials. 2020 Jun 29;21:592. doi: 10.1186/s13063-020-04532-7 (PMC7325031; doi:10.1186/s13063-020-04532-7)
Supplement: Supplementary file 2 — Additional file 2. Study consent form. [file 13063_2020_4532_MOESM2_ESM.pdf]

camh
Centre for Addiction and Mental Health

Study Information and Consent Form

Study Title
Personalized dosing of nicotine replacement for smoking cessation: an effectiveness randomized placebo-controlled trial.

Investigators:

Principal Investigators: Peter Selby, MBBS 416-535-8501 x77432
Laurie Zawertailo, PhD 416-535-8501 x 77422
Person to Contact About Research:
Dr. Laurie. Zawertailo 416-535-8501 x 77422

You are being asked to participate in a randomized controlled research study. The study will be conducted at the Centre for Addiction and Mental Health and the University of Ottawa Heart Institute. The study is funded by the Canadian Cancer Society. Approximately 500 people (men and women) will take part in this study.

Purpose of the Study:
To determine if adjusting the nicotine patch dose to match an individual's needs is a safe and effective way of helping an individual quit smoking over 12 weeks of treatment and maintaining it for up to 12 months.

Procedures:
If you volunteer for the study, you will be asked to first complete an assessment involving a physical examination, psychiatric interview, and a blood sample (one 10ml vial). This appointment will take 2 to 3 hours of your time. You will be asked questions about your medical history, family history, mental health history, substance use, alcohol use, use of tobacco, readiness to stop smoking, and other questions related to your general well-being. There are no right or wrong answers to the questions and you do not have to answer a question, if that is your choice. All blood samples will be taken by trained personnel who are experienced in this procedure.

If you are a woman of child-bearing age, we will ask you to confirm that you are not pregnant, nor likely to be pregnant, for the duration of the study. You will be asked to provide a urine sample for a pregnancy test.

If you meet the study's eligibility criteria, you will begin participating in the study.

Study Visits:
This study will involve 7 to 9 weekly study appointments at the Nicotine Dependence Clinic (NDC) at CAMH (175 College St., Toronto). On weeks where you do not have a schedule in-person study visit, a telephone check-in will be conducted.

At each clinic appointment:

- We will test for signs of smoking (using a Smokerlyzer breath test which measures the carbon monoxide in your system).
- You will complete study questionnaires assessing your desire to smoke and any physical and emotional changes that may be occurring.
- You will receive smoking cessation counselling as part of the treatment.
- You will visit the study physician who will prescribe your nicotine patches.
- You will also be required to return any unused medication from the time of your previous in-person visit as well as all of your used patches.

The clinic visits will take about 30 minutes.

During this baseline visit, you will be given a 2 week supply of patches. You will start wearing your first patch the next day. You will continue to wear a new patch once a day for the next 2 weeks. If you are able to remain completely smoke-free for the final week of this two week period, you will continue to receive the standard 21mg nicotine patch for the remainder of the study (10 more weeks). If you are not able to remain smoke-free during this time, you will be assigned at random to either additional nicotine patches or additional placebo patches. So you will have a fifty-fifty chance to either use patches with nicotine or patches without any nicotine (placebo) in addition to your 21mg Nicoderm patch. Your patch dose will be adjusted weekly (depending on how many cigarettes you are still smoking) for the next 5 weeks or until you are able to stop smoking cigarettes. It is important to note that the maximum approved dose for nicotine patch is 21mg per day. If assigned to the active nicotine patch group, you may exceed this dose.

During the treatment phase you will not be able to use any other smoking cessation medications or nicotine replacement products (including e-cigarettes).

At the week 10 appointment you will be asked to provide another blood sample.

At the week 12 appointment you will be asked to provide a urine sample for analysis of a chemical called anabasine that is only present in the urine of tobacco smokers.

You will receive $10 upon completing each in-person study visit. You will also be provided with 2 TTC tokens to pay for your transportation.

After the 12 week treatment period, you will receive a package of nicotine patches for your tapering down period. You may be contacted by phone weekly during the tapering down phase to see how you are doing and to answer a few questions.

You will be invited to return to the Nicotine Dependence Clinic for follow-up appointments at 6- and 12-months after you started the study. You will be asked questions about your smoking behaviour since your last appointment and you will breathe into the Smokerlyzer. You will receive $25 and 2 TTC tokens for completing each of these appointments. If you have relapsed to smoking, you will be offered treatment at the Nicotine Dependence Clinic.

Study Medication:
All study participants will receive 21mg nicotine patches. The patches used for the adjusted doses will contain either active nicotine or placebo (non-active). Neither you nor the study personnel will know which you will be given because the patches will be identical. Only the CAMH pharmacist will know. You will be given the medication to take every day for 12 weeks of the treatment period and a package of NRT for your tapering down period.

Risks:
The most common side effect associated with the use of the nicotine patch is a temporary redness and/or burning sensation at the site where the patch is applied. This side effect was reported in about 47% of nicotine patch users. Among nicotine patch users, 3% reported swelling at the location of the patch and 2% experienced an allergic skin rash in response to the patch. Additional side effects of the nicotine patch include headaches (15.9%), insomnia (15.7%), dizziness (7.1%), abnormal dreams (6.3%), indigestion (5.8%), nausea (5.4%), and weakness (5.1%).

You will be followed very closely by the study personnel for any changes in your mood and health. Any significant new findings developed during the course of the research, which may relate to your willingness to continue participation, will be addressed in a timely manner.

Benefits:
The nicotine patch combined with in-person counselling is the most-effective treatment for smoking cessation. Participating in this study will increase your chances of quitting successfully. The knowledge gained from this study may be used to improve current smoking cessation treatment.

Confidentiality:
Your answers to the study questions are confidential to the extent permitted by law and will be available only to the study investigators. A copy of this consent form and clinical information obtained during your assessment and visits with your health care professional will be placed in your health record.

As part of the Research Services Quality Assurance Program, this study may be monitored and/or audited by a member of the Quality Assurance Team. Your research records and CAMH records may be reviewed during, which confidentiality will be maintained as per CAMH policies and to the extent permitted by law.

As a part of continuing review of the research, your study records may be assessed on behalf of the Research Ethics Board. A person from the research ethics team may contact you (if your contact information is available) to ask you questions about the research study and your consent to participate. The person assessing your file or contacting you must maintain your confidentiality to the extent permitted by law. Your personal information will not be made available to anyone else without a court order or your written permission. Any reports or publications based on this study will not mention your name or identify you in any way.

This study is under the authority of Health Canada because it involves the use of nicotine patch doses that are higher than the approved dose of 21mg per day. Your records may therefore be assessed by the Health Canada Therapeutic Products Programme.

A description of this clinical trial will be available on http://www.ClinicalTrials.gov as required by U.S. Law. This website will not include information that can identify you. At most, the website will include a summary of the results. You can search this website at any time.

Refusal to participate:
You can choose not to participate in this study. If you choose not to participate, the Research staff will discuss alternative smoking cessation options that are available to you.

Withdrawal from research:
Your participation is completely voluntary and you may decline to participate or withdraw from this research at any time. This will not affect your current or future treatment at CAMH. If you choose to withdraw the information that you have provided up to the point of withdrawal will be kept for future analyses. The study investigators may also terminate your participation in the study if they feel that you are not fulfilling the requirements of the study.

Contacts:
If you have any further questions or desire further information about this study, you may contact Dr. Laurie Zawertailo at 416-535-8501, ext. 77422. If you have any questions about your rights as a study participant, you may contact Dr. Robert Levitan, Chair of the Research Ethics Board, Centre for Addiction and Mental Health, at 416-535-8501, ext. 34020. In the event of a research related injury contact: Dr. Peter Selby 416-535-8501 ext. 77432.

AGREEMENT TO PARTICIPATE

I, ___________________________________have read (or had read to me) the consent form for the study named **Personalized dosing of nicotine replacement for smoking cessation: an effectiveness randomized placebo-controlled trial.**

- The researcher or a member of the research staff has discussed with me the risks of participation in this study.
- I have read all the information in the Study Information Sheet and had time to think about the information, and all of my questions have been answered to my satisfaction.
- I voluntarily agree to participate in this research study, to follow study procedures, and to provide necessary information to the researcher as requested.
- I am under no pressure to participate in this study, and I understand that I may withdraw from the study at any time. I understand that my participation in the study may be terminated by the study investigators/ researchers if deemed necessary.
- Any significant new finding developed during the course of the research, which may relate to my willingness to continue participating, will be provided to me in a timely manner.
- By signing this consent form, I am not giving up my legal rights or releasing the investigators, researchers, or sponsors from their legal and professional obligations.
- A copy of this consent form and clinical information obtained during my assessment and visits with my health care professional will be placed in my health record.
- I will receive a copy of this signed consent form.
- I consent to participate in the study including the laboratory investigation.

Participant Complete:

________________________	________________________
Print First Name	Print Last Name
________________________	________________________
Signature	Date (DD-MMM-YYYY)

Person Obtaining Consent: My signature below means that the participant freely consented to enroll in the research described above. The participant: was fully informed about the study including procedures, risks and benefits; read the consent form or had it read to them; was given the opportunity to have questions answered prior to signing the informed consent document; agreed to comply with program procedures including follow-up contact and was given a copy of the informed consent document.

________________________	________________________	
Print First Name	Print Last Name	
________________________	________________________	_____________
Signature	Date (DD-MMM-YYYY)	Time
