# Supplementary material for: Personalized dosing of nicotine replacement therapy versus standard dosing for the treatment of individuals with tobacco dependence: study protocol for a randomized placebo-controlled trial
Source: Trials. 2020 Jun 29;21:592. doi: 10.1186/s13063-020-04532-7 (PMC7325031; doi:10.1186/s13063-020-04532-7)
Supplement: Supplementary file 3 — Additional file 3. Genetics sub-study consent form. [file 13063_2020_4532_MOESM3_ESM.pdf]

camh
Centre for Addiction and Mental Health

Genetics Sub-study Information and Consent Form

Study Title

Personalized dosing of nicotine replacement for smoking cessation: an effectiveness randomized placebo-controlled trial.

Investigators:

Principal Investigator: Peter Selby, MBBS 416-535-8501 ext. 77432

Co- Principal Investigator: Laurie Zawertailo, PhD 416-535-8501 ext. 77422

Person to Contact About Research: Dr. Laurie. Zawertailo 416-535-8501 ext. 77422

You are being asked to participate in an experimental research study. This study will be conducted at the Centre for Addiction and Mental Health (CAMH, 175 College St., Toronto), under the supervision of Drs. Selby and Zawertailo. Up to 500 people (men and women) will take part in this study.

Background and Purpose of the Study

As part of the main study entitled “Personalized dosing of nicotine replacement for smoking cessation: an effectiveness randomized placebo-controlled trial”, you will be prescribed nicotine patches for smoking cessation. The efficacy of this treatment method varies among individuals as a result of genetic variations, some of which lead to differing rates of nicotine breakdown, while others affect the way your body and brain respond to nicotine, or otherwise affect your ability to quit smoking.

We would like to explore how genetic variation among people receiving nicotine patches alters their response to treatment. We can see if your ability to break down the nicotine is normal, too fast, or too slow by looking at your DNA. We will also look at your DNA to see if we can find other changes that may affect your ability to quit smoking.

What will I be asked to do if I agree to take part in the genetics component of the study?

If you agree to enroll in this part of the study, we will ask you to provide a blood sample (approximately 10ml) for DNA testing. You may decide to take part in the genetics sub-study at any point during the study.

Are there any risks?

There is a slight risk of bruising or inflammation at the site of venipuncture for the blood sample.

A risk of genetic research is the possibility of disclosure of your study participation or research results to individuals not involved in the study, such as insurers or employers. Dr. Zawertailo's team will take all reasonable steps to protect your research information in order to minimize the potential of harm to you from an unintended disclosure of genetic or clinical information.

In the event that you suffer injury as a direct result of participating in this study, normal legal rules on compensation will apply. By signing this consent form, you are in no way waiving your legal rights or releasing the investigators from their professional and legal responsibilities.

What are the benefits to me?

The information collected in this study may help to advance our knowledge of how genetic make-up influences the response to nicotine replacement patches. In the future, this knowledge may improve the effectiveness of this treatment method by identifying factors that influence response to treatment.

What will happen to my sample and my medical information?

We will work with and store your sample securely for an indefinite period of time. We will require anyone holding your sample to hold the research information and any results in confidence so that they are not divulged to a third party without our approval.

Is my participation voluntary? What happens if I no longer wish to take part in this study?

Taking part in this study is entirely voluntary. You may decide not to take part or you may decide to take part and then change your mind. This will not affect your participation in the main study. You can withdraw from this study at any time without giving a reason. Also, it will not affect your access to current or future treatment at CAMH. If you withdraw from this study, your sample will be destroyed. However, we will keep any genetic results and clinical information collected up to that point.

Can I be excluded from the study?

You are being asked to participate in the genetics component of the study because you have qualified for the main study. In special cases, your sample may not be used and will be destroyed. This might occur if the study is stopped for other reasons.

Will I benefit financially from the study?

You will receive $25 in cash for participating in this sub-study at the end of the study visit at which the sample is collected.

Will my personal information be kept confidential?

We will not give your genetic results to anyone, unless required by law. “Anyone” includes you, your family, your insurance company, and your employer. Your genetic results are for research purposes only and have no established use for clinical diagnosis or treatment. Although your sample and information are coded, we cannot guarantee that a connection between you and your results will not be established.

To protect your information, you will be assigned a study code. This number will be used to keep track of your samples and medical information. All information that we collect from you and the results from your sample analysis will not identify you in any way. The file containing the link between the study code and your name will be stored on a secure server and password protected. Only the study investigators and delegates will have access to this file.

Your name will not appear in any publications or external reports about this research. Also, your medical information and any coded results will be entered on a computer and stored in an electronic database on an encrypted server. We will comply with the relevant laws to protect the confidentiality of research participants when processing and storing personal information.

We may collaborate with other research organizations in other locations, including commercial companies, who may want to use your sample and already collected medical information for studying genetic material and substances related to research on addictive or psychiatric disorders. Your name or any other information that could identify you will not be released. We will require that other collaborators keep your anonymized medical information confidential.

As part of continuing review of the research, your study records may be assessed on behalf of the Research Ethics Board. A person from the research ethics team may contact you (if your contact information is available) to ask you questions about the research study and your consent to participate. The person assessing your file or contacting you must maintain your confidentiality to the extent permitted by law.

This study is under the authority of Health Canada as it involves evaluating the use of nicotine patches at unapproved doses. Your records may therefore be assessed by the Health Canada Therapeutic Products Programme.

As part of the Research Services Quality Assurance Program, this study may be monitored and/or audited by a member of the Quality Assurance Team. Your research records and CAMH records may be reviewed during which confidentiality will be maintained as per CAMH policies and extent permitted by law.

Contacts:

If you have any further questions or desire further information about this study, you may contact Dr. Laurie Zawertailo at 416-535-8501, ext. 77422. If you have any questions about your rights as a study participant, you may contact Dr. Robert Levitan, chair of the Research Ethics Board, Centre for Addiction and Mental Health, at 416-535-8501, extension 34020.

Centre for Addiction and Mental Health

AGREEMENT TO PARTICIPATE

I, ______________________________________have read (or had read to me) the consent form for the study named **Personalized dosing of nicotine replacement for smoking cessation: an effectiveness randomized placebo-controlled trial – Genetics Sub study.** This study is for research purposes only.

- I voluntarily agree to take part in this study.
- I have read this informed consent form and had the opportunity to ask about anything I do not understand. I am satisfied with the answers I have been given.
- I have been given time to consider whether or not to take part in this research.
- I am aware that I am free to withdraw from the study at any time and that this withdrawal would not affect my future medical treatment.
- Information will be treated in the strictest confidence. By signing and dating this consent form I agree that ethics committees/ institutional review boards can and will access my medical records for research purposes.
- I agree to my sample being used in this study and in any future research.
- I will receive a copy of this signed consent form.

I consent to participate in the study.

Participant Name: ________________________________
please print

Participant Signature: ______________________________ Date: __________________________

Person who conducted informed consent discussion:

Signature: ____ Date: __________________________

Name: ____
please print
